# Dexmedetomidine versus haloperidol for sedation of non-intubated patients with hyperactive delirium during the night in a high dependency unit: study protocol for an open-label, parallel-group, randomized controlled trial (DEX-HD trial)

**DOI:** 10.1186/s12871-023-02158-1

**Published:** 2023-06-03

**Authors:** Takuma Minami, Hirotoshi Watanabe, Takao Kato, Kaori Ikeda, Kentaro Ueno, Ai Matsuyama, Junya Maeda, Yoji Sakai, Hisako Harada, Akira Kuriyama, Kyohei Yamaji, Naoki Kitajima, Jun Kamei, Yudai Takatani, Yuki Sato, Yugo Yamashita, Toshiyuki Mizota, Shigeru Ohtsuru

**Affiliations:** 1 Department of Primary Care and Emergency Medicine, Graduate School of Medicine, Kyoto University, 54 Kawahara-cho, Shogoin, Sakyo-ku, Kyoto 606-8507 Japan; 2 Department of Cardiovascular Medicine, Graduate School of Medicine, Kyoto University, 54 Kawahara-cho, Shogoin, Sakyo-ku, Kyoto 606-8507 Japan; 3 Institute for Advancement of Clinical and Translational Science (iACT), Kyoto University Hospital, 54 Kawahara-cho, Shogoin, Sakyo-ku, Kyoto 606-8507 Japan; 4 Department of Diabetes, Endocrinology and Nutrition, Graduate School of Medicine, 54 Kawahara-cho, Shogoin, Sakyo-ku, Kyoto 606-8507 Japan; 5 Department of Biomedical Statistics and Bioinformatics, Graduate School of Medicine, Kyoto University, 54 Kawahara-cho, Shogoin, Sakyo-ku, Kyoto 606-8507 Japan; 6 Department of Nursing, Kyoto University Hospital, 54 Kawahara-cho, Shogoin, Sakyo-ku, Kyoto 606-8507 Japan; 7 Department of Clinical Pharmacology and Therapeutics, Kyoto University Hospital, 54 Kawahara-cho, Shogoin, Sakyo-ku, Kyoto 606-8507 Japan; 8 Department of Anesthesia, Kyoto University Hospital, 54 Kawahara-cho, Shogoin, Sakyo-ku, Kyoto 606-8507 Japan

**Keywords:** Agitation, Delirium, Haloperidol, Dexmedetomidine, Randomized controlled trial, Pharmacotherapy, Intubation, Sedatives, Hospital units

## Abstract

**Background:**

Delirium is common in critically ill patients. Haloperidol has long been used for the treatment of delirium. Dexmedetomidine has recently been used to treat delirium among intubated critically ill patients. However, the efficacy of dexmedetomidine for delirium in non-intubated critically ill patients remains unknown. We hypothesize that dexmedetomidine is superior to haloperidol for sedation of patients with hyperactive delirium, and would reduce the prevalence of delirium among non-intubated patients after administration. We will conduct a randomized controlled trial to compare dexmedetomidine and haloperidol for the treatment of nocturnal hyperactive delirium in non-intubated patients in high dependency units (HDUs).

**Methods:**

This is an open-label, parallel-group, randomized controlled trial to compare the efficacy and safety of dexmedetomidine and haloperidol for nocturnal hyperactive delirium in non-intubated patients at two HDUs of a tertiary hospital. We will recruit consecutive non-intubated patients who are admitted to the HDU from the emergency room, and allocate them in a 1:1 ratio to the dexmedetomidine or haloperidol group in advance. The allocated investigational drug will be administered only when participants develop hyperactive delirium (Richmond Agitation-Sedation Scale [RASS] score ≥1 and a positive score on the Confusion Assessment Method for the ICU between 19:00 and 6:00 the next day) during the night at an HDU. Dexmedetomidine is administered continuously, while haloperidol is administered intermittently. The primary outcome is the proportion of participants who achieve the targeted sedation level (RASS score of between -3 and 0) 2h after the administration of the investigational drug. Secondary outcomes include the sedation level and prevalence of delirium on the day following the administration of the investigational drugs, and safety. We plan to enroll 100 participants who develop nocturnal hyperactive delirium and receive one of the two investigational drugs.

**Discussion:**

This is the first randomized controlled trial to compare the efficacy and safety of dexmedetomidine and haloperidol for sedation of non-intubated critically ill patients with hyperactive delirium in HDUs. The results of this study may confirm whether dexmedetomidine could be another option to sedate patients with hyperactive delirium.

**Trial registration:**

Japan Registry of Clinical Trials, jRCT1051220015, registered on 21 April 2022.

## Background

Delirium is acute mental disturbance and cognitive impairment, and frequently occurs in critically ill patients [1–4]. Delirium is associated with mortality, prolonged length of hospital stay, and increased risk of dementia [5–8], and also an increment in burden to medical staffs [9, 10]. Particularly, hyperactive delirium accompanied with agitation and restlessness could cause patients to fall from a bed or accidentally remove indwelling catheters and other life-supporting devices [8, 11], and prompt control is required in critically ill patients. Therefore, treatment of delirium has been an important issue in the critical care setting.

Haloperidol has long been used as the standard treatment for hyperactive delirium [12, 13], but it is associated with high failure rates in patients with hyperactive delirium [14, 15] or did not reduce the duration of delirium [16]. Dexmedetomidine is a highly selective α2 adrenergic receptor agonist, and has been used to prevent or treat hyperactive delirium in recent years. It has been reported that the time taken to achieve an optimal sedation level with dexmedetomidine may be shorter than that with haloperidol [17]. Furthermore, recent meta-analyses have reported that dexmedetomidine is more effective than other drugs including haloperidol in reducing the duration of delirium in patients in intensive care units (ICUs) [18]. From a pharmacological viewpoint, the half-life of dexmedetomidine (1.8-3.1 h) is shorter than that of haloperidol (18-54h) [13, 19, 20]. Thus, dexmedetomidine is administered by continuous infusion, while haloperidol is administered intermittently, as done in a recent landmark trial [16, 20]. Accordingly, we anticipate that dexmedetomidine will be easier to titrate and will exert its effect earlier than haloperidol, and might be preferable for sedation of patients with hyperactive delirium.

In Japan, the number of ICU beds is relatively small (7.3 beds per 100,000 population in Japan) [21], compared to that in other developed countries (e.g., 33.6 beds per 100,000 population in the United States) [22]. Consequently, some critically ill patients in Japan are treated in a high dependency unit (HDU), which is termed a “step-down unit” in the United States and “high care unit” in Japan. Since the majority of patients in HDUs are not intubated, high-dose sedative agents cannot be easily administered due to the risk of respiratory depression. Additionally, the number of nurses in HDUs is lower than that in ICUs in Japan (generally, the nurse:patient ratio is 1:4 in HDUs compared to 1:2 in ICUs). Thus, it is difficult to take adequate care of patients with hyperactive delirium in HDUs. To date, one report showed that dexmedetomidine was effective for sedation of non-intubated patients in the ICU with hyperactive delirium that was refractory to haloperidol [15]. However, the efficacy of dexmedetomidine has rarely been examined in non-intubated critically ill patients with hyperactive delirium in HDUs.

We hypothesize that continuous dexmedetomidine use is superior to intermittent haloperidol use for hyperactive delirium in non-intubated patients in HDUs in terms of rapid sedation and reducing the duration of delirium. Consequently, this study aimed to test that dexmedetomidine achieves the targeted sedation level more rapidly than haloperidol. Additionally, we will examine some potentially clinically relevant outcomes such as the amount of drug needed for adequate maintenance of the targeted sedation level, prevalence of delirium on the day following administration of the treatment, and contents of nursing care.

## Methods

### Study design, ethics, and trial registration

The present study is an investigator-initiated, open-label, parallel-group, randomized controlled trial to compare the efficacy and safety of dexmedetomidine and haloperidol for hyperactive delirium in non-intubated patients at two HDUs of a tertiary care hospital. The Ethics Committee of Kyoto University Graduate School and Faculty of Medicine approved this study. The study protocol has been prepared in compliance with the SPIRIT 2013 Statement [23]. This trial is registered at the Japan Registry of Clinical Trials [https://jrct.niph.go.jp/] (No. jRCT1051220015).

### Participants

#### Inclusion criteria

Our institution, Kyoto University Hospital, is a 1,141-bed tertiary care hospital. It has several HDUs; this study will be conducted at two HDUs (one for the Department of Primary Care and Emergency Medicine [EHDU] and the other for the Department of Cardiovascular Medicine [CHDU]). We will approach all consecutive, non-intubated adults (aged ≥ 20 years) who are hospitalized from the emergency room to one of these HDUs. There will be no maximum age limit nor gender limit. Participants in the EHDU are expected to have endogenous diseases such as infectious diseases and abnormal electrolytes; patients with mild trauma who do not require emergent surgery with general anesthesia are also expected. Participants in the CHDU are expected to have cardiovascular diseases such as acute coronary syndrome, acute decompensated heart failure, pulmonary thromboembolism, and Stanford B aortic dissection. While both HDUs can occasionally accommodate patients who had undergone surgery with general anesthesia, those who are extubated before enrollment in our study will be eligible for this study.

#### Exclusion criteria

Patients who fulfill one or more of the following will be excluded:

- Patients who have received non-invasive ventilation (NIV) on admission to the HDU.
- Patients with tracheostomy.
- Patients who have already started to receive dexmedetomidine or haloperidol by bolus injection, drip infusion, or intramuscular injection before admission to the HDU.
- Patients with a diagnosis of schizophrenia or mania.
- Patients with contraindications to haloperidol or dexmedetomidine (e.g., allergy to any of the investigational drugs, prolonged QTc on electrocardiogram)
- Pregnant or possibly pregnant women and those lactating.
- Patients who cannot communicate in the Japanese language.
- Patients who are judged to be inappropriate for this study by the principal investigator or by those who are deemed to be qualified by the principal investigator.

### Enrollment of participants and allocation

#### Consent to participate, enrollment of participants

**Fig. 1:**
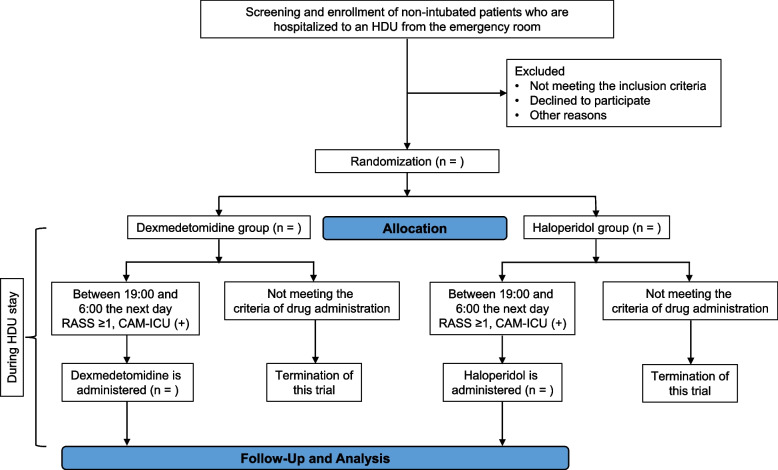
Flowchart of enrollment of participants, and populations of analysis. CAM-ICU = Confusion Assessment Method for the ICU; RASS = Richmond Agitation-Sedation Scale

We will require written informed consent by patients or their authorized surrogates to be included in this trial. Doctors among the investigators will attempt to ask all eligible patients fulfilling the above criteria to participate in this trial, irrespective of the presence of delirium before enrollment (Fig. 1). There are two main reasons for obtaining consent in advance in this manner. First, we would like our patients by themselves to give consent for participation in the trial before developing delirium, because delirious patients may not clearly show their will. In cases where patients cannot state their own will due to severe dementia or impaired consciousness, for example, consent by an authorized surrogate such as a relative will be recognized. Second, we will attempt to get consent beforehand so that the investigational drug can be administered immediately if the participant develops hyperactive delirium.

#### Allocation

In this trial, participants will be stratified by the EHDU or CHDU. First, a statistician who is not involved in this trial made an allocation list based on a computer-generated random number table using a 1:1 ratio of dexmedetomidine or haloperidol for each HDU before initiation of the trial. Second, a secretary who is not involved in this trial will conceal the names of the allocated drugs within the lists with un-stickable tapes, and distribute the respective allocation lists to each HDU before enrollment of the first patient in the trial. Finally, when consent for trial participation is obtained from a participant, the physician will write besides the identification number of the participant’s electronic health record, age, gender, and primary disease on the list in sequential order before unsealing the tapes to avoid allocation again and confirm their allocation.

### Protocols of investigational drug administration

The detailed administration protocols of each investigational drug are as follows (Fig. 1 and Table 1). Nurses in each HDU routinely measure the Richmond Agitation-Sedation Scale (RASS) [24] and Confusion Assessment Method for the ICU (CAM-ICU) [25] at 10:00 and 22:00. In this trial, when a participant shows obvious agitation between 19:00 and 6:00 the next day during their HDU stay, nurses additionally measure the RASS and CAM-ICU, and consider whether to start administration of the investigational drug based on the protocol described below. We will not restrict continuation of hypnotics that had been taken by the patient before admission, or prophylactic use of hypnotics and oral antipsychotic agents except for the investigational drugs. Furthermore, we will allow an attempt of drug administration based on the protocols only once on the first night when a participant meets our criteria below, and we will not restrict the type of post-investigation treatment on any night.

**Table 1:**
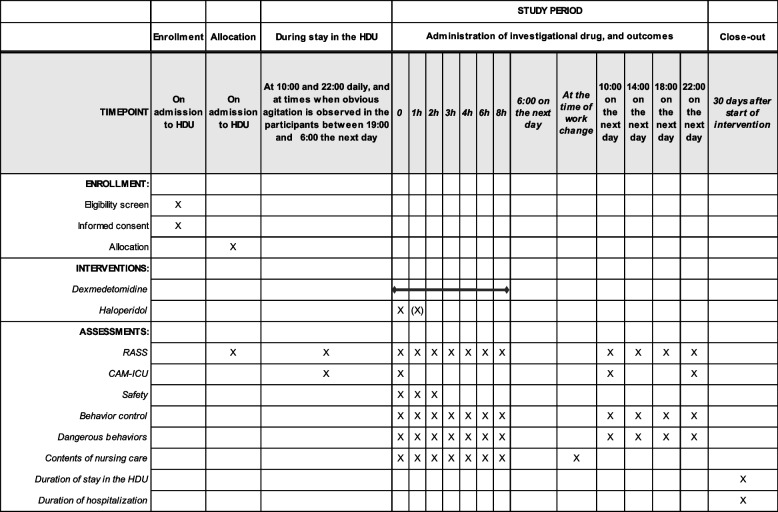
The schedule of enrollment, interventions, and summary of outcomes in this trial

*Abbreviations: CAM-ICU* Confusion Assessment Method for the ICU, *HDU* high dependency unit, *RASS* Richmond Agitation-Sedation Scale

#### Dexmedetomidine group

When a participant first attains a RASS score ≥1 and a positive score on the CAM-ICU between 19:00 and 6:00 the next day during the participant’s stay in the HDU, infusion of dexmedetomidine (DEXMEDETOMIDINE INTRAVENOUS SYRINGE; Nipro, Osaka, Japan) will be started at 0.3 to 0.7 mcg/kg/h and will be maintained at 0.1 to 0.7 mcg /kg/h to keep the RASS score at between -3 and 0 until 6:00 the next day (Table 1). A loading dose of ≤6 mcg/kg/h for 10 minutes is permitted if the participant shows much agitation such as a RASS score of 3 to 4.

If a RASS score of between -3 and 0 cannot be achieved after initiation of dexmedetomidine, rescue administration of other drugs including haloperidol is permitted at the discretion of a physician. We will attempt to postpone starting rescue administration of other drugs within 2 hours from the initiation of dexmedetomidine administration as much as possible. However, if the participant is in imminent harm due to hyperactive delirium, rescue administration of other drugs can be started within the 2-hour period. In addition, dexmedetomidine can be interrupted or resumed at any time for hypotension and bradycardia. When continuously administering an intravenous rescue drug, we will set the dose to reach a target RASS score of between -3 and 0, and also continue the rescue drug until 6:00 the next day.

#### Haloperidol group

When a participant first attains a RASS score ≥1 and a positive score on the CAM-ICU between 19:00 and 6:00 the next day during the participant’s stay in the HDU, haloperidol (Serenace Injection; Sumitomo pharma, Osaka, Japan) 2.5 mg will be administered by bolus injection or drip infusion for 30 minutes or by intramuscular injection (Table 1). If the participant does not achieve a RASS score ≤0 within 1 hour from the first administration of haloperidol, haloperidol 2.5 mg will be additionally administered by bolus injection or drip infusion for 30 minutes or by intramuscular injection (Table 1). The route of administering haloperidol will be determined at the discretion of treating physicians and nurses.

If a RASS score ≤0 cannot be achieved after the second administration of haloperidol within 2 hours from the first administration of haloperidol, rescue administration of other drugs including dexmedetomidine is permitted at the discretion of a physician. We will attempt to postpone starting rescue administration of other drugs within 2 hours from the first administration of haloperidol as much as possible. However, if the patient is in imminent harm due to hyperactive delirium, rescue administration of other drugs can be started within the 2-hour period. When continuously administering an intravenous rescue drug including dexmedetomidine, we will set the dose to reach a target RASS score of between -3 and 0, and also continue administration of the rescue drug until 6:00 the next day.

### Outcomes

Following is the summary of outcomes in the trial (Table 1).

#### Primary outcome

- Proportion of participants who achieve the targeted sedation level (RASS score of between -3 and 0) at 2 hours after the start of administration of the investigational drug

#### Secondary outcomes

- Time to achieve a RASS score of between -3 and 0 after the start of administration of the investigational drug
- RASS scores at 1, 2, 3, 4, 6 and 8 hours after the start of administration of the investigational drug
- Proportion of participants with a RASS score of between -3 and 0 at 1, 2, 3, 4, 6, and 8 hours after the start of administration of the investigational drug
- Duration that the RASS score is between -3 and 0 within the 8-hour period from the start of administration of the investigational drug to 8 hours
- Proportion of participants with delirium on the day following the start of administration of the investigational drug
- RASS score on the day following the start of administration of the investigational drug
- Number or proportion of delirium-free days during the stay in the HDU
- Duration of stay in the HDU, and duration of the admission
- Proportion of participants who require rescue administration of another drug(s) after administration of the investigational drug
- Safety (oversedation, hypotension, bradycardia, transition to advanced respiratory support such as NIV or invasive mechanical ventilation, and so on)
- Behavior control (e.g., restraint mittens, physical restraint)
- Dangerous behaviors
- Contents of nursing care
- Incidence of delirium as defined below among all participants

### Measurement of outcomes

#### RASS, CAM-ICU, and classification of delirium and agitation

We classified delirium and agitation by the RASS score [24] and CAM-ICU result [25] as follows: hyperactive delirium, RASS score ≥1, and CAM-ICU (+) [26]; hypoactive delirium, RASS score between -3 and 0, and CAM-ICU (+) [26]; mixed delirium, presenting hyperactive delirium and hypoactive delirium on the same day; agitation, RASS score ≥1 regardless of the CAM-ICU result. As described above, the nurses in our HDUs regularly measure the RASS score and CAM-ICU every 12 hours, and additionally measure the RASS score and CAM-ICU while participants are presenting agitation between 19:00 and 6:00 the next day during their HDU stay. Furthermore, nurses will measure the RASS score at 1, 2, 3, 4, 6, and 8 hours after the initiation of administration of the investigational drug to examine the sedation level as control of hyperactive delirium. We further measure the RASS score at 10:00, 14:00, 18:00, and 22:00, and CAM-ICU at 10:00 and 22:00 to check whether the participants have agitation or unintentional sedation, and whether the participants have hypoactive delirium, hyperactive delirium, or mixed delirium on the day following the start of administration of the investigational drug. In our HDUs, the nurses routinely evaluate RASS and CAM-ICU and are familiar with the measurement. Additionally, we made sure that the nurses appropriately checked the measurement of the RASS and CAM-ICU by holding a lecture meeting, before initiating the present trial.

#### Safety

We define each safety issue when one or more of the following respective criteria are met.

- Hypotension: presence of a novel systolic blood pressure ≤90 mmHg; newly initiating a vasopressor; discontinuing or interrupting the investigational drug due to hypotension within 2 hours from the start of administration of the investigational drug.
- Bradycardia: a novel heart rate ≤50 /min; initiating positive chronotropic agents; initiating temporary pacing; changing modes or settings of the permanent pacemaker; discontinuing or interrupting administration of the investigational drug due to bradycardia within 2 hours from the start of administration of the investigational drug.
- Oversedation: a RASS score of between -4 and -5 for 8 hours from the start of administration of the investigational drug
- Transition to advanced respiratory support as escalation to NIV or invasive mechanical ventilation
- Unintentional diurnal sedation with a RASS score ≤-1 on the next day after stopping administration of the investigational drug. (If continuous sedative drugs including dexmedetomidine are not discontinued on the next day, we did not judge unintentional diurnal sedation).

#### Behavior control, dangerous behaviors, and contents of nursing care

We will measure the duration of behavior control (nurses watching the participant at bedside, use of mitten gloves, use of physical restraint, and use of bed-leaving sensor), and will observe the participants for dangerous behaviors such as accidental removal of indwelling catheter and other devices, falls, and violence from participants from the time of start of administration of the investigational drug to the next day. To investigate the burden of nursing care, we will examine the duration of nursing care at the participant’s bedside as measured by a stopwatch (ud0010; Molten Corporation, Hiroshima, Japan) during the 8-hour period from the start of administration of the investigational drug. The stopwatch is a large stationary type with high visibility of measuring time so that nurses will remember to start and stop the stopwatch by pressing a button. We will place two of the stopwatches in front of the participant’s room, and concurrently measure the duration of nursing care provided by up to 2 nurses. In particular, for night-shift nurses, we further asked two of them in charge of the participants to answer whether they felt burdened by the night work, whether they could fully take a rest, and whether they ended their workday on time.

### Data collection and management

#### Plans for assessment and collection of outcomes

Trained study staff will collect clinical data on the participants from their electronic medical charts. Outcomes described above such as RASS, CAM-ICU, delirium, safety, behavior control, dangerous behavior, and contents of nursing care will be collected by the treating nurses from the time of start of administration of the investigational drug to the next day with a trial-specific paper-based form. Data on the duration of stay in the HDU, duration of the admission, and incidences of all types of delirium were also collected by the trained staff from the electronic health charts.

#### Plans to promote participant retention and complete follow-up

We have no serious concern about participant retention because all data will be obtained during the hospitalization.

#### Data management

All such data will be stored in an electronic file. Entered data will be confirmed by a second individual for accuracy. Only individuals who have permission from the principal investigator will have access to the database. We will give a study identification number for each participant in the electronic file for anonymity. The table that shows a list of the identification number for this study and for the corresponding participant’s electronic health record, respectively, will be stored in locked cabinets in a secure area.

#### Data monitoring

Since this trial is small-scaled and conducted at a single center, we will not establish a data monitoring committee.

### Statistics

#### Statistical analyses

We will express variables as means (standard deviations), medians [interquartile range], or number (%). We will use the Student’s t-test, analysis of variance, Mann-Whitney test, or the Kruskal-Wallis test to compare continuous variables as appropriate, and the chi-square test to compare categorical variables. We will define the full analysis set (FAS) as participants who are enrolled in this study and receive any investigational drug at least once; and the per protocol set (PPS) as those who are enrolled in this study and receive an allocated investigational drug at least once as planned in the administration protocol. The primary outcome is the proportion of participants who achieve the target RASS score (-3 to 0) at 2 hours after the start of administration of the investigational drug in the FAS. We will also analyze the proportion of participants who achieve the target RASS score (-3 to 0) at 2 hours after administration of the investigational drug in the PPS, and other outcomes in the FAS and PPS, respectively. Two-sided P-values of <.05 will be considered statistically significant. We will perform analyses using SPSS software, version 25 (SPSS, Inc, Chicago, IL).

#### Sample size estimation

In this trial, the sample size is the number of participants who develop hyperactive delirium and receive an investigational drug according to the study protocol, because the primary outcome is the proportion of participants who achieve the targeted sedation level at 2 hours after the start of administering the investigational drug. A previous meta-analysis showed that approximately 90% of hyperactive delirium patients were successfully treated with dexmedetomidine [18], although the patients who were included in the meta-analysis were not limited to non-intubated patients. In contrast, approximately 65% of non-intubated patients with hyperactive delirium were adequately treated with haloperidol [15]. However, the participants in the above meta-analysis were possibly treated with other sedatives because the original studies included mechanically-ventilated patients [18], and the dose of haloperidol in that study was higher than that in our trial [15]. Therefore, in our protocol using a lone investigational drug, we underestimated the efficacy of dexmedetomidine or haloperidol by approximately 5%; we used the estimated efficacy in the dexmedetomidine and haloperidol groups of 85% and 60%, respectively, to calculate the sample size. With two-sided alpha values of 0.05 and a power of 80%, 49 participants will be required for each group (98 in total). Assuming a dropout rate of 2% due to the detection of a serious protocol violation later, we decided to enroll 100 participants. The sample size calculation was performed with Stata version 17.0 (StataCorp, College Station, TX).

### Criteria for withdrawal

Participants who meet any of the following conditions will be withdrawn from the study:

- Participants who do not meet the criteria for initiating administration of an investigational drug during their HDU stay, and subsequently do not receive any investigational drug (Fig. 1).
- Participants who receive any investigational drug before meeting the criteria for starting administration of an investigational drug.
- Participants who request to be removed from the study or who withdraw consent.

### Adverse event reporting and harms

Adverse events are defined as all undesirable or non-intended illnesses and signs that may have a potential causal association with the investigational drugs. All adverse events are shared with the investigators, and will be dealt with appropriately. Serious adverse events related to this trial will be reported to the Kyoto University Certified Review Board (CRB) within 15 days.

### Plans for communicating important protocol amendments to relevant parties

Any modification to the protocol will be discussed and agreed to by the study investigators, and will be subsequently submitted to the Kyoto University CRB for approval. We will also update the protocol at the Japan Registry of Clinical Trials.

## Discussion

This is the first randomized controlled trial to compare the efficacy and safety of dexmedetomidine and haloperidol for sedation of non-intubated critically ill patients with hyperactive delirium during the night in HDUs. If a favorable effect and safety of dexmedetomidine for hyperactive delirium in non-intubated patients are shown, dexmedetomidine would be a promising treatment option for sedation of critically ill patients with hyperactive delirium in HDUs and ICUs.

### Trial status

Patient recruitment began in May 2022. The trial was originally expected to end in March 2023, but we estimate that patient recruitment will be completed in March 2024 due to slow accrual. The current protocol is version 1.4, dated 1 March 2023.

### Dissemination

We will present the results of the trial at national and/or international conferences and in peer-reviewed journals.

## Data Availability

The dataset generated from the current study will be available from the corresponding author upon reasonable request.

## Abbreviations

CAM-ICU: Confusion Assessment Method for the ICU
HDU: High dependency unit
ICU: Intensive care unit
RASS: Richmond Agitation-Sedation Scale

## References

[1] Inouye SK, Rushing JT, Foreman MD, Palmer RM, Pompei P (1998). Does delirium contribute to poor hospital outcomes? A three-site epidemiologic study. J Gen Intern Med..

[2] Bergeron N, Dubois MJ, Dumont M, Dial S, Skrobik Y (2001). Intensive Care Delirium Screening Checklist: evaluation of a new screening tool. Intensive Care Med..

[3] Ely EW, Shintani A, Truman B, Speroff T, Gordon SM, Harrell FE (2004). Delirium as a predictor of mortality in mechanically ventilated patients in the intensive care unit. JAMA..

[4] Ely EW, Girard TD, Shintani AK, Jackson JC, Gordon SM, Thomason JWW (2007). Apolipoprotein E4 polymorphism as a genetic predisposition to delirium in critically ill patients. Crit Care Med..

[5] González M, Martínez G, Calderón J, Villarroel L, Yuri F, Rojas C (2009). Impact of delirium on short-term mortality in elderly inpatients: a prospective cohort study. Psychosomatics..

[6] Marcantonio ER, Kiely DK, Simon SE, John Orav E, Jones RN, Murphy KM (2005). Outcomes of older people admitted to postacute facilities with delirium. J Am Geriatr Soc..

[7] Davis DHJ, Muniz Terrera G, Keage H, Rahkonen T, Oinas M, Matthews FE (2012). Delirium is a strong risk factor for dementia in the oldest-old: a population-based cohort study. Brain..

[8] O’Regan NA, Fitzgerald J, Timmons S, O’Connell H, Meagher D (2013). Delirium: a key challenge for perioperative care. Int J Surg..

[9] Schmitt EM, Gallagher J, Albuquerque A, Tabloski P, Lee HJ, Gleason L (2019). Perspectives on the Delirium Experience and Its Burden: Common Themes Among Older Patients, Their Family Caregivers, and Nurses. Gerontologist..

[10] Mossello E, Lucchini F, Tesi F, Rasero L (2020). Family and healthcare staff’s perception of delirium. Eur Geriatr Med..

[11] Pun BT, Ely EW (2007). The importance of diagnosing and managing ICU delirium. Chest..

[12] Celis-Rodríguez E, Birchenall C, de la Cal MÁ, Castorena Arellano G, Hernández A, Ceraso D (2013). Clinical practice guidelines for evidence-based management of sedoanalgesia in critically ill adult patients. Med intensiva..

[13] Barr J, Pandharipande PP. The pain, agitation, and delirium care bundle: synergistic benefits of implementing the 2013 Pain, Agitation, and Delirium Guidelines in an integrated and interdisciplinary fashion. Crit Care Med. 2013;41(9 Suppl 1):S99–115. 10.1097/CCM.0b013e3182a16ff0 23989099

[14] Skrobik YK, Bergeron N, Dumont M, Gottfried SB (2004). Olanzapine vs haloperidol: treating delirium in a critical care setting. Intensive Care Med..

[15] Carrasco G, Baeza N, Cabré L, Portillo E, Gimeno G, Manzanedo D (2016). Dexmedetomidine for the Treatment of Hyperactive Delirium Refractory to Haloperidol in Nonintubated ICU Patients: A Nonrandomized Controlled Trial. Crit Care Med..

[16] Andersen-Ranberg NC, Poulsen LM, Perner A, Wetterslev J, Estrup S, Hästbacka J, et al. Haloperidol for the Treatment of Delirium in ICU Patients. N Engl J Med. 2022;387(26):2425–35. 10.1056/NEJMoa2211868 36286254

[17] Reade MC, O’Sullivan K, Bates S, Goldsmith D, Ainslie WRSTJ, Bellomo R. Dexmedetomidine vs. haloperidol in delirious, agitated, intubated patients: a randomised open-label trial. Crit Care. 2009;13:R75. 10.1186/cc7890 PMC2717438 19454032

[18] Liu X, Xiong J, Tang Y, Gong C-C, Wang D-F (2021). Role of dexmedetomidine in the treatment of delirium in critically ill patients: a systematic review and meta-analysis. Minerva Anestesiol..

[19] Jacobi J, Fraser GL, Coursin DB, Riker RR, Fontaine D, Wittbrodt ET (2002). Clinical practice guidelines for the sustained use of sedatives and analgesics in the critically ill adult. Crit Care Med..

[20] Dotson B (2016). Comparing Dexmedetomidine With Haloperidol for the Treatment of Hyperactive Delirium in Nonintubated ICU Patients. Crit Care Med..

[21] Phua J, Faruq MO, Kulkarni AP, Redjeki IS, Detleuxay K, Mendsaikhan N (2020). Critical Care Bed Capacity in Asian Countries and Regions. Crit Care Med..

[22] Halpern NA, Goldman DA, Tan KS, Pastores SM (2016). Trends in Critical Care Beds and Use Among Population Groups and Medicare and Medicaid Beneficiaries in the United States: 2000–2010. Crit Care Med..

[23] Chan AW, Tetzlaff JM, Gøtzsche PC, Altman DG, Mann H, Berlin JA (2013). SPIRIT 2013 explanation and elaboration: guidance for protocols of clinical trials. BMJ..

[24] Ely EW, Truman B, Shintani A, Thomason JWW, Wheeler AP, Gordon S (2003). Monitoring sedation status over time in ICU patients: reliability and validity of the Richmond Agitation-Sedation Scale (RASS). JAMA..

[25] Ely EW, Inouye SK, Bernard GR, Gordon S, Francis J, May L (2001). Delirium in mechanically ventilated patients: validity and reliability of the confusion assessment method for the intensive care unit (CAM-ICU). JAMA..

[26] Card E, Pandharipande P, Tomes C, Lee C, Wood J, Nelson D (2015). Emergence from general anaesthesia and evolution of delirium signs in the post-anaesthesia care unit. Br J Anaesth..

